# Language comprehension in mild cognitive impairment: a bibliometric study of research trends and thematic structures

**DOI:** 10.3389/fpsyg.2026.1886461

**Published:** 2026-08-26

**Authors:** Yao Fan, Lin Guo, Lin Fan, Jiaosheng Qiu

**Affiliations:** 1 School of Foreign Languages, Taishan University, Tai’an, Shandong, China; 2 School of English and International Studies, Beijing Foreign Studies University, Beijing, China; 3 National Research Centre for Foreign Language Education, Beijing Foreign Studies University, Beijing, China

**Keywords:** mild cognitive impairment, language comprehension, cognitive aging, bibliometric analysis, VOSviewer, CiteSpace

## Abstract

**Objective:**

A decline in language comprehension may represent a potential indicator of progressive cognitive deterioration in people with neurodegenerative diseases, and this sign often becomes noticeable as early as the mild cognitive impairment (MCI) stage. This study aimed to map the knowledge domain of language comprehension research in MCI, summarize its major research patterns, and identify recent research fronts, with a view to informing early identification, assessment, and intervention in neurodegenerative diseases.

**Methods:**

A bibliometric approach was adopted. Relevant publications were retrieved from the Web of Science (WoS) Core Collection, with the time span set from 2001 to 2025. After refining, manual checking, and deduplication, 914 articles and reviews were included. VOSviewer and CiteSpace were used to analyze the publication trends, subject categories, country collaborations, co-cited references, keyword co-occurrence, co-citation clusters, and citation bursts. Scopus was used as an independent database to assess the robustness of the WoS-based findings, and a generalized additive model (GAM) was fitted to compare the annual publication trajectories derived from the two databases.

**Results:**

Research on language comprehension in MCI showed an overall increase within the study period. The WoS and Scopus datasets differed in overall publication volume but showed no statistically detectable difference in the shape of their annual publication trends. The field is mainly distributed across neurosciences, clinical neurology, geriatrics & gerontology, psychology, and linguistics. The USA plays a leading role in both publication numbers and international collaborations. The knowledge foundation of this field is largely grounded in the clinical characterization of MCI, Alzheimer’s disease (AD) diagnostic frameworks, and biomarker research. Major research topics are organized around the clinical and biomarker-based framing of MCI and AD, together with discourse, connected speech, semantic processing, and cognitive mechanisms of comprehension. Recent hotspots point to early language markers, ecologically valid measures, and automated approaches to detection and assessment.

**Conclusion:**

This study offers a macroscopic view of language comprehension research in MCI. Future work should pay more attention to stage-related changes, cross-linguistic variations, clearer distinctions among comprehension processes, and more reliable measures for clinical monitoring and intervention evaluation. Future reviews may also extend this work to language production and broader communicative functioning in MCI.

## 1 Introduction

With the continuous increase in people’s life expectancy and the global expansion of the elderly population, along with declining birth rates, age-related issues have drawn more and more attention. As one of the most common age-related issues, neurodegenerative dementia is an important cause of concern worldwide, because it is clinically established to be irreversible in nature (World Health Organization, 2025). Among all types of dementia, Alzheimer’s disease (AD) is often described as the most common form, because it accounts for about 60–70% of all dementia cases (World Health Organization, 2025). The clinical manifestation of AD usually progresses through an intermediate stage of mild cognitive impairment (MCI). At this stage, subtle cognitive changes occur before obvious dementia symptoms appear, making MCI an important window for identifying early cognitive markers within the neurodegenerative disease continuum (Petersen et al., 2014).

The term MCI was introduced by Reisberg et al. in the 1980s. It was used to describe an intermediate stage that closely matches Stage 3 on the Global Deterioration Scale (GDS) (Reisberg et al., 1982). Flicker et al. (1991) later used this concept in an empirical study of mildly cognitively impaired older adults. Petersen et al. (2014) reviewed prior definitions of MCI and described it as an intermediate clinical state. Many studies view MCI as a transitional phase between cognitive changes in normal aging and the more typical impairments seen in dementia (Petersen et al., 2014). Earlier clinical descriptions also emphasized two points that are still widely used in current research. One is that cognitive decline can be reflected through objective assessment, such as decline in memory (Petersen et al., 1999). The other is that people remain largely independent in everyday life, even though subtle difficulties may already appear in some individuals (Gauthier et al., 2006; Petersen et al., 1999). Although episodic memory decline is traditionally viewed as a typical indicator of MCI, cognitive changes often extend to other domains, such as executive function and language. In the present study, language is understood in a broad contemporary sense, including lexical, syntactic, and semantic processing as well as discourse, pragmatics, and everyday communication. Although the present review focuses specifically on comprehension, language changes in MCI also involve expressive abilities, including lexical retrieval, verbal fluency, and connected speech. Comprehension and production are therefore treated as related but distinct aspects of language functioning.

Within this broader framework of language functioning, the present study focuses specifically on language comprehension because of its important role in supporting successful communication and its potential sensitivity to early cognitive decline. Language comprehension requires readers or listeners to integrate linguistic and contextual information. This process is not independent. It draws on cognitive resources like attention, working memory, and executive control. Because these resources are vulnerable in aging and neurodegenerative conditions, language comprehension tasks may help to detect subtle cognitive changes before more obvious functional decline appears. In this context, studies have started to examine the language comprehension processes in MCI, rather than regarding language as a single test variable (e.g., Chapman et al., 2002; Drummond et al., 2015; Kokje et al., 2022; Taler and Phillips, 2008).

Previous research on language comprehension in MCI and related at-risk states is usually conducted within the domain of amnestic MCI (aMCI), in which the decline of memory is an obvious feature. For individuals with MCI, even though their lexical access and comprehension of simple sentences are relatively preserved, language comprehension difficulties emerge when tasks require continuous integration or inference processing. At the sentence level, individuals in subjective cognitive decline (SCD), a preclinical at-risk state that may precede MCI, have been found to perform more poorly on sentences with noncanonical word order, although their general cognitive ability is still tested as normal (López-Higes et al., 2025). Their poorer performance may be interpreted by the mechanism of working memory, as working memory can influence the association between cognitive reserve and sentence comprehension (López-Higes et al., 2024). At the discourse and text levels, language comprehension difficulties are more prominent. Understanding discourse requires the maintenance of the situation model, ongoing semantic integration, and generation of inferences. Individuals with MCI often struggle with these inferential processes (Maziero et al., 2021) and show reduced sensitivity to non-literal meanings, like metaphors, indicating weakened semantic integration in figurative comprehension (Cardoso et al., 2014; Yang and Huang, 2023). These deficits are even more obvious in ecologically valid tasks, such as narrative and pragmatic comprehension. Narrative comprehension in aMCI is frequently characterized by incomplete narrative structure and irrelevant micropropositions (Drummond et al., 2015). Their pragmatic comprehension reveals similar patterns. Individuals with aMCI have been found to perform less accurately when comprehension depends on social-cognitive inference, such as understanding verbal irony (Gaudreau et al., 2013). This kind of difficulty is not only about formal decoding and meaning integration. It also reflects problems in the inferential processes that support successful comprehension in everyday communication.

A handful of reviews have attempted to synthesize language-related findings in MCI and related conditions. An early and influential narrative review by Taler and Phillips (2008) compared language performance in MCI and AD across different types of language tasks, including standardized and non-standardized tests. They found that language deficits are not restricted to AD but can be detected as early as during the MCI stage. However, the review focused on overall language performance and provided only limited analyses of deficits in language comprehension or the specific cognitive demands involved in comprehension. Mueller et al. (2018) systematically reviewed connected speech in MCI and AD using picture description tasks. They concluded that picture description is sensitive to language changes in many aspects of AD, and that some of these changes have potential value for MCI diagnosis. Although Mueller et al. (2018) focused primarily on language production rather than comprehension, their findings remain relevant to the present review. Their review suggests that connected speech and language comprehension rely on overlapping semantic, executive, and discourse-level processes. Accordingly, production-based measures provide complementary evidence that language change in MCI extends beyond receptive processing alone. More recently, Kokje et al. (2022) conducted a systematic review of discourse comprehension as a potential early behavioral marker of AD and MCI. Across the included studies, participants with mild AD and MCI usually performed worse on discourse comprehension than cognitively healthy groups. However, the results were not always consistent, especially when the outcomes came from comprehension questions. The review also showed clear differences in task materials, scoring criteria, and performance measures, which made it difficult to compare findings across studies or to relate specific characteristics to patterns of cognitive impairment.

The existing studies suggest that language-related findings are increasingly regarded as potential behavioral markers of early cognitive changes in pre-dementia stages. This focus is not merely theoretical. Detection of language-related changes may support earlier risk identification and earlier intervention, which could help delay symptom onset and slow down daily functional decline. However, much of the research still concentrates on a limited set of tasks or domains, and many reviews remain largely qualitative. More importantly, systematic and quantitative reviews that focus specifically on language comprehension in MCI remain relatively few. There is still a lack of macroscopic and data-driven analyses of this field, which limits our understanding of how related topics have evolved over time.

To fill this gap, the present study conducts a scientometric and bibliometric analysis of publications on language comprehension in MCI. The purpose of this study is to map the knowledge domain of language comprehension research in MCI, provide an overview of its current status and development, and clarify how related research is involved in the broader study of cognitive changes in neurodegenerative diseases. To be specific, the study attempts to address the following questions: (1) How has research on language comprehension in MCI developed over time, and what publication patterns, disciplinary distribution, and collaboration patterns characterize this field? (2) What are the main knowledge foundations and thematic structures of this field, as reflected in the reference co-citation network, keyword co-occurrence network, and reference co-citation clusters? And (3) what recent and ongoing research fronts can be identified from citation bursts, and what do they reveal about the current development of this field?

## 2 Methods

### 2.1 Data collection

In the current study, the relevant articles were searched and retrieved from the Web of Science (WoS) Core Collection through the Clarivate WoS platform on January 1, 2026. The database included the Science Citation Index Expanded (SCIE), Social Sciences Citation Index (SSCI), Arts and Humanities Citation Index (A&HCI), Conference Proceedings Citation Index-Science (CPCI-S), and Conference Proceedings Citation Index-Social Science and Humanities (CPCI-SSH). The data set was obtained using the following search query:

TS = ((“mild cognitive impairment” OR “mild neurocognitive disorder” OR MCI) AND (sentence* OR discourse OR text* OR narrative* OR pragmatic* OR inferen* OR metaphor* OR irony OR semantic*) AND (comprehension OR comprehend* OR understanding OR understand* OR processing OR process* OR interpret* OR integration)).

The publication date was set from 2001-01-01 to 2025-12-31. Initially, 1,156 records were retrieved from the WoS Core Collection. Then the dataset was further refined with the following two restrictions: (a) only articles written in English were collected; (b) only research articles and reviews were included, with book reviews excluded. The remaining records were manually checked to confirm their relevance to language comprehension in MCI. Then, duplicate records were removed using CiteSpace. Finally, 914 qualified records were included in the present study.

For manual relevance screening, titles and abstracts were initially screened by the first author according to the predefined eligibility criteria. The corresponding author subsequently reviewed the inclusion decisions and all records for which eligibility was uncertain. Full texts were consulted when relevance could not be determined from the title and abstract. Any disagreements were resolved through discussion until consensus was reached. Records were included if they focused on language comprehension in MCI. Detailed eligibility criteria for manual relevance screening are presented in Supplementary Table S5.

**FIGURE 1:**
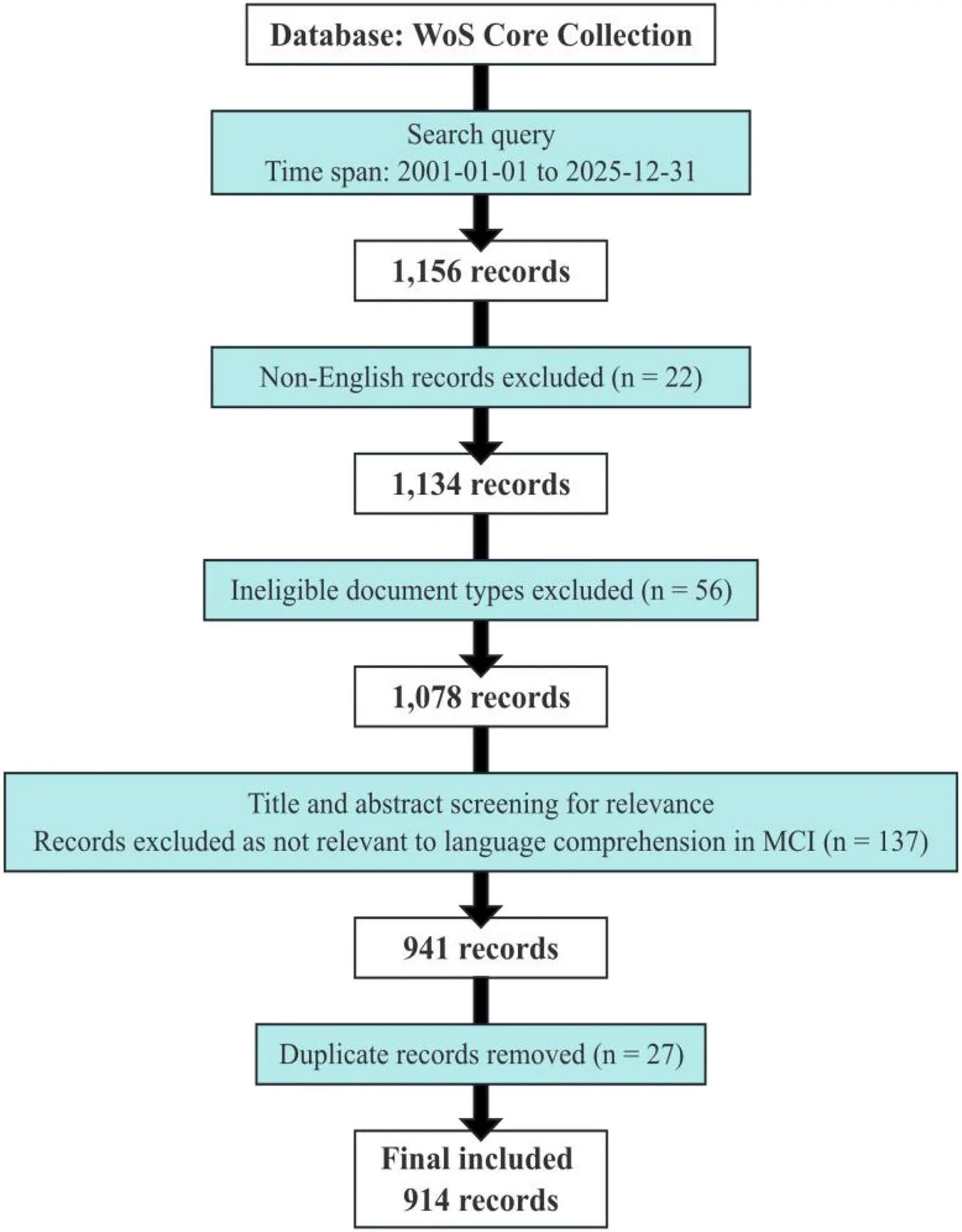
Flowchart of data retrieval and screening procedures.

To improve transparency and reproducibility (PRISMA-inspired), the key information on data retrieval and screening procedures was demonstrated in Figure 1. Because this study is a bibliometric mapping review rather than an intervention-based systematic review, risk-of-bias assessment was not applicable.

Records were exported separately in formats compatible with CiteSpace and VOSviewer. For CiteSpace, WoS records were exported as plain text files with “Full Record and Cited References.” For VOSviewer, the records were exported as tab delimited files with the same record content. Because of the export limits of the database interface, the records were downloaded in sequential files. Detailed export information is provided in Supplementary Table S2.

### 2.2 Bibliometric analysis and visualization

The publications retrieved from the database were visualized and analyzed using VOSviewer (version 1.6.19) and CiteSpace (version 6.4.R1). The annual publication number was quantitatively analyzed and demonstrated in a line chart to visualize the general research trend of the field in the past 25 years. VOSviewer (van Eck and Waltman, 2010) was used to visualize collaborating countries, co-cited references, and co-occurring keywords, so as to reveal the relationships among them and the knowledge foundation of this field. In the maps generated by VOSviewer, each node represents an analyzed item, node size reflects its frequency, and the thickness of the links indicates the strength of co-authorship, co-citation, or co-occurrence relationships. Items shown in the same color were assigned to the same cluster.

Different inclusion thresholds were applied according to the type of network. The minimum thresholds for country co-authorship, reference co-citation, and keyword co-occurrence analyses, together with the number of items meeting each threshold, are reported in Supplementary Table S3. Full counting and association-strength normalization were used in the VOSviewer analyses. Other layout and clustering settings were retained at their default values.

CiteSpace (Chen, 2006) was used to analyze co-occurring subject categories, reference co-citation clustering, and citation bursts in the collected records. The co-occurring subject categories were used to show the disciplinary composition and interdisciplinary nature of the field. The clustering analysis was used to identify the thematic structure of the literature, while citation burst detection was used to reflect emerging research trends. For the CiteSpace maps, each node also represents an analyzed item, and node size indicates its frequency. Links between nodes represent the relationships among them, and the colors of nodes and links correspond to different time slices, with cooler colors indicating earlier years and warmer ones indicating more recent years. For the clustering map generated by CiteSpace, each cluster represents a major research topic in the field. These analyses provided complementary perspectives on the disciplinary distribution, international collaboration patterns, knowledge base, thematic structure, and research frontiers of language comprehension research in MCI. They also helped place this field within the broader study of cognitive changes in neurodegenerative diseases.

CiteSpace analyses covered the period from 2001 to 2025 using 1-year time slices. Subject-category analysis used “Category” as the node type, whereas reference co-citation clustering and citation-burst analysis used “Reference.” The node-selection criterion, pruning options, cluster-labeling method, and burst-detection settings are reported in Supplementary Table S4.

The analytical workflow was designed to support both transparent reporting and careful interpretation of the bibliometric outputs. In line with recent methodological recommendations (Arsyi et al., 2026), the database selection, search and screening procedures, export formats, inclusion thresholds, software settings, and analytical parameters were documented in detail. The complete search strategies, data-export information, VOSviewer thresholds, CiteSpace parameters, eligibility criteria, and additional model specifications are provided in Supplementary material.

### 2.3 Validation analysis using Scopus

To assess the robustness of the WoS-based findings, an additional validation analysis was conducted using Scopus. The Scopus search was performed on March 12, 2026, using the same search strategy, time span, language restriction, document types, and inclusion criteria to maximize consistency with the WoS dataset. The complete Scopus search string and retrieval settings are provided in Supplementary Table S1. The final Scopus dataset contained 509 records. The records were exported in CSV format for analysis in VOSviewer; detailed export information is provided in Supplementary Table S2.

The Scopus dataset was analyzed separately and was not merged with the WoS dataset. Cross-database validation covered four dimensions: annual publication trends, productive journals, productive countries and collaboration patterns, and frequent keywords and thematic structures. The purpose was to determine whether the principal publication and thematic patterns identified in WoS were broadly reproduced in another major multidisciplinary database, rather than to establish statistical equivalence between the two databases. Cross-database comparison has been widely used to assess the robustness of bibliometric findings (Archambault et al., 2009).

To formally compare the annual publication trajectories derived from WoS and Scopus, a Poisson generalized additive model (GAM) was fitted in R (version 4.6.1) using the *mgcv* package (version 1.9–4). Annual publication count was modeled using a Poisson distribution with a log link. Database was included as a categorical predictor, and year was represented by a penalized thin-plate regression spline. WoS was treated as the reference database, and an ordered-factor difference smooth was included to test whether the temporal trajectory for Scopus differed from that for WoS. The basis dimension was set to *k* = 8, and smoothing parameters were estimated using restricted maximum likelihood. A negative-binomial model was fitted to examine possible overdispersion, and alternative basis dimensions of *k* = 6 and *k* = 10 were examined in sensitivity analyses. Model adequacy was evaluated using convergence diagnostics, basis-dimension checks, residual plots, and residual autocorrelation functions. The additional GAM specifications are provided in Supplementary Table S6 and Supplementary Figures S1, S2.

## 3 Results

### 3.1 Annual publication trend, productive journals, and category distribution

**FIGURE 2:**
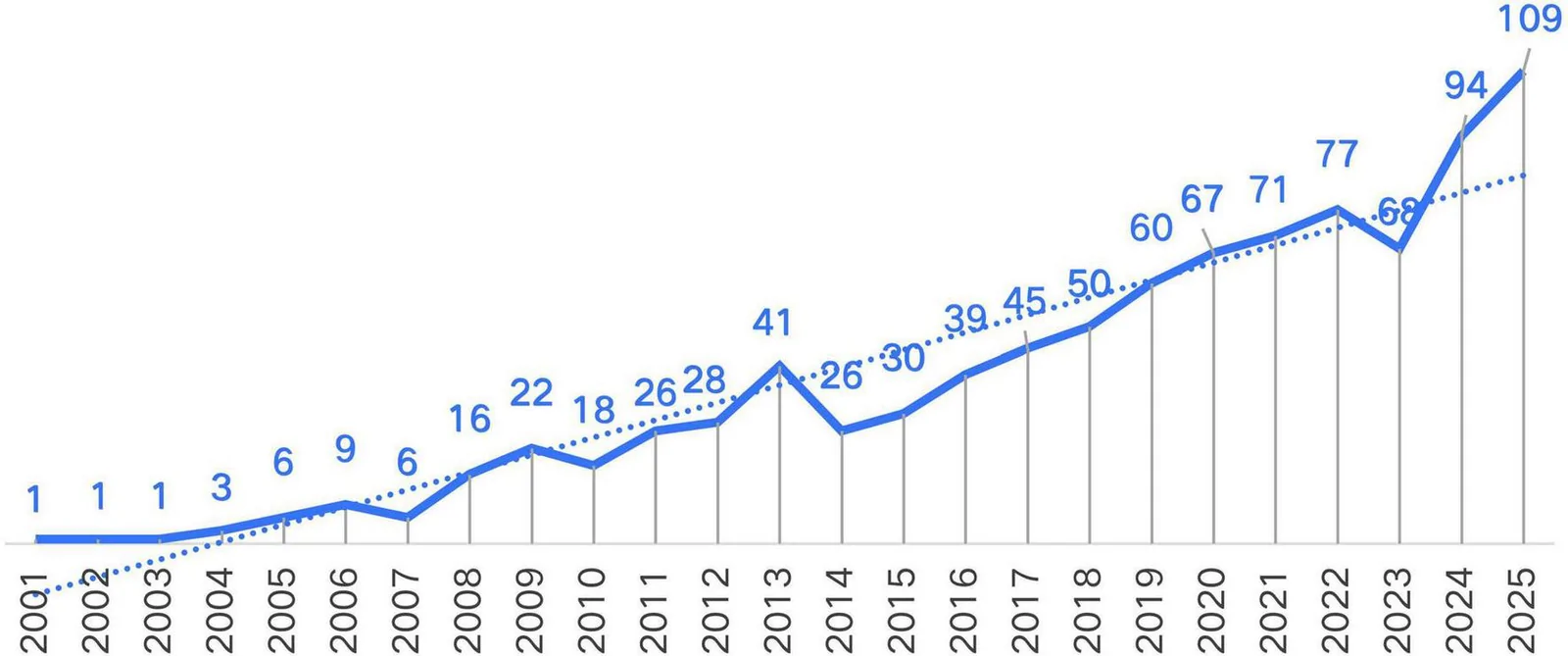
Numbers of annual publications in WoS core collection (2001–2025).

From 2001 to 2007, only 1 to 9 papers were published annually. Over the following 15 years, annual publication output increased overall, although year-to-year fluctuations remained. Annual output increased more sharply after 2022, and the highest annual count in the dataset was recorded in 2025, with 109 publications. Figure 2 shows an overall upward trend from 2001 to 2025. The trend of annual publications suggests that research on language comprehension in MCI has gradually become an active topic over the past 25 years.

The collected articles are distributed in 200 journals, while a limited number of key journals contributed the majority of articles analyzed in this research. *Journal of Alzheimer’s Disease* is the most productive journal in this domain with 57 articles, followed by *Cortex*, *Neuropsychology*, *Frontiers in Aging Neuroscience*, *Neuropsychologia*, *Ageing Research Reviews*, *Current Alzheimer Research*, *Journal of the International Neuropsychological Society*, *Frontiers in Neurology*, and *Journal of Clinical and Experimental Neuropsychology*. These journals collectively reflect the interdisciplinary nature of this field. Table 1 lists the top ten productive journals.

**TABLE 1:** The 10 most productive journals (2001–2025).

Rank	Title of journals	Number of published articles
1	Journal of Alzheimer’s Disease	57
2	Cortex	20
3	Neuropsychology	19
4	Frontiers in Aging Neuroscience	18
5	Neuropsychologia	16
6	Ageing Research Reviews	15
7	Current Alzheimer Research	14
8	Journal of the International Neuropsychological Society	14
9	Frontiers in Neurology	13
10	Journal of Clinical and Experimental Neuropsychology	13

**FIGURE 3:**
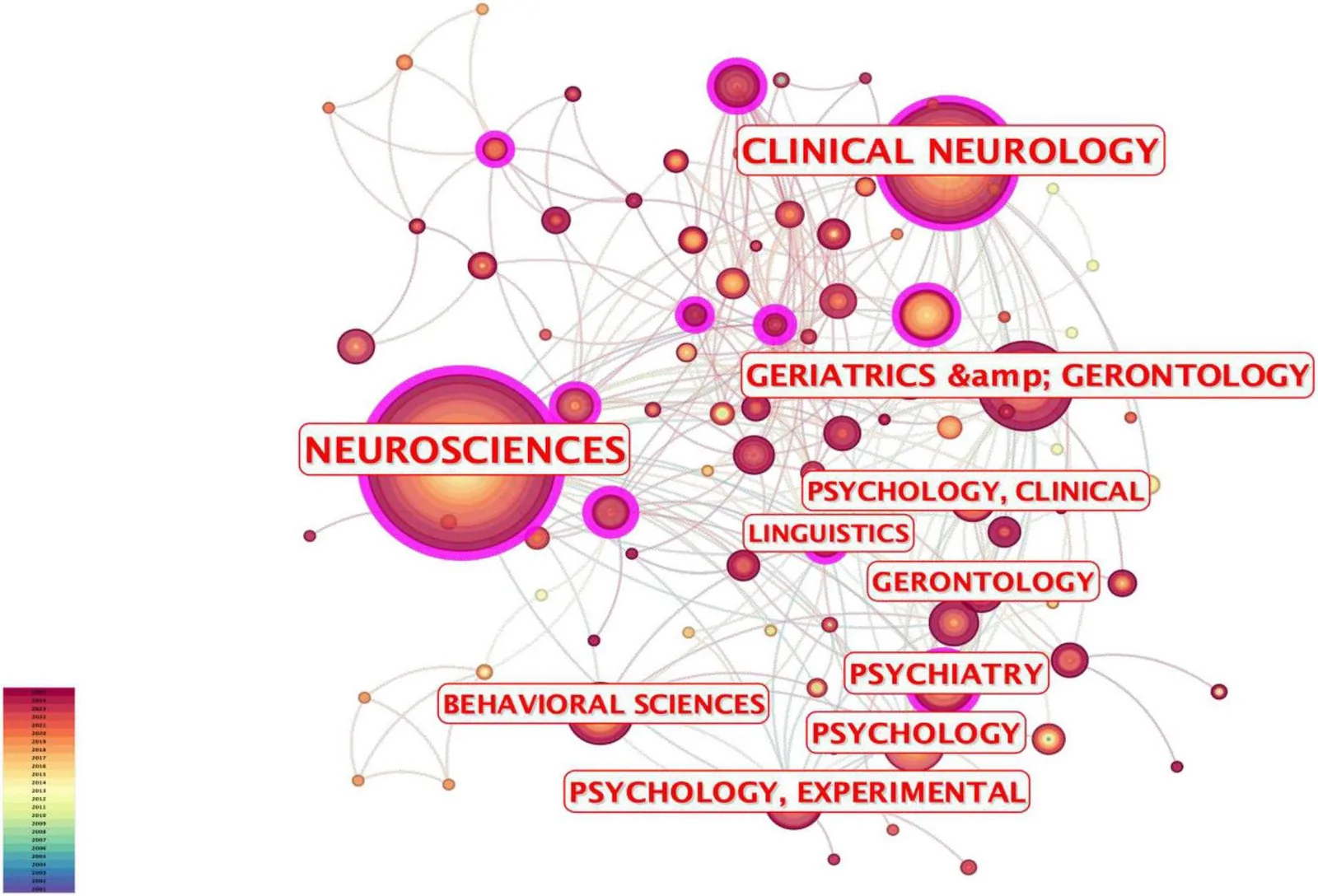
The major co-occurring subject categories (2001–2025).

Analysis of subject categories can further demonstrate the interdisciplinary nature of this field. As shown in Figure 3, the most prominent categories are neurosciences, clinical neurology, geriatrics & gerontology, psychology, clinical, psychology, experimental, behavioral sciences, and linguistics. This pattern indicates that the field is primarily dominated by neuroscience and clinical research on cognitive impairment and neurodegeneration. This area also obtained substantial attention from psychology, behavioral science, aging-related disciplines, and linguistics. This indicates that the field is not limited to medical or neurological description, and it also incorporates cognitive, behavioral, and linguistic perspectives. The appearance of linguistics as a distinct category suggests that language-related problems are not merely treated as clinical symptoms, but are examined as a distinct dimension of cognitive change.

### 3.2 Geographical distribution and collaboration patterns

**FIGURE 4:**
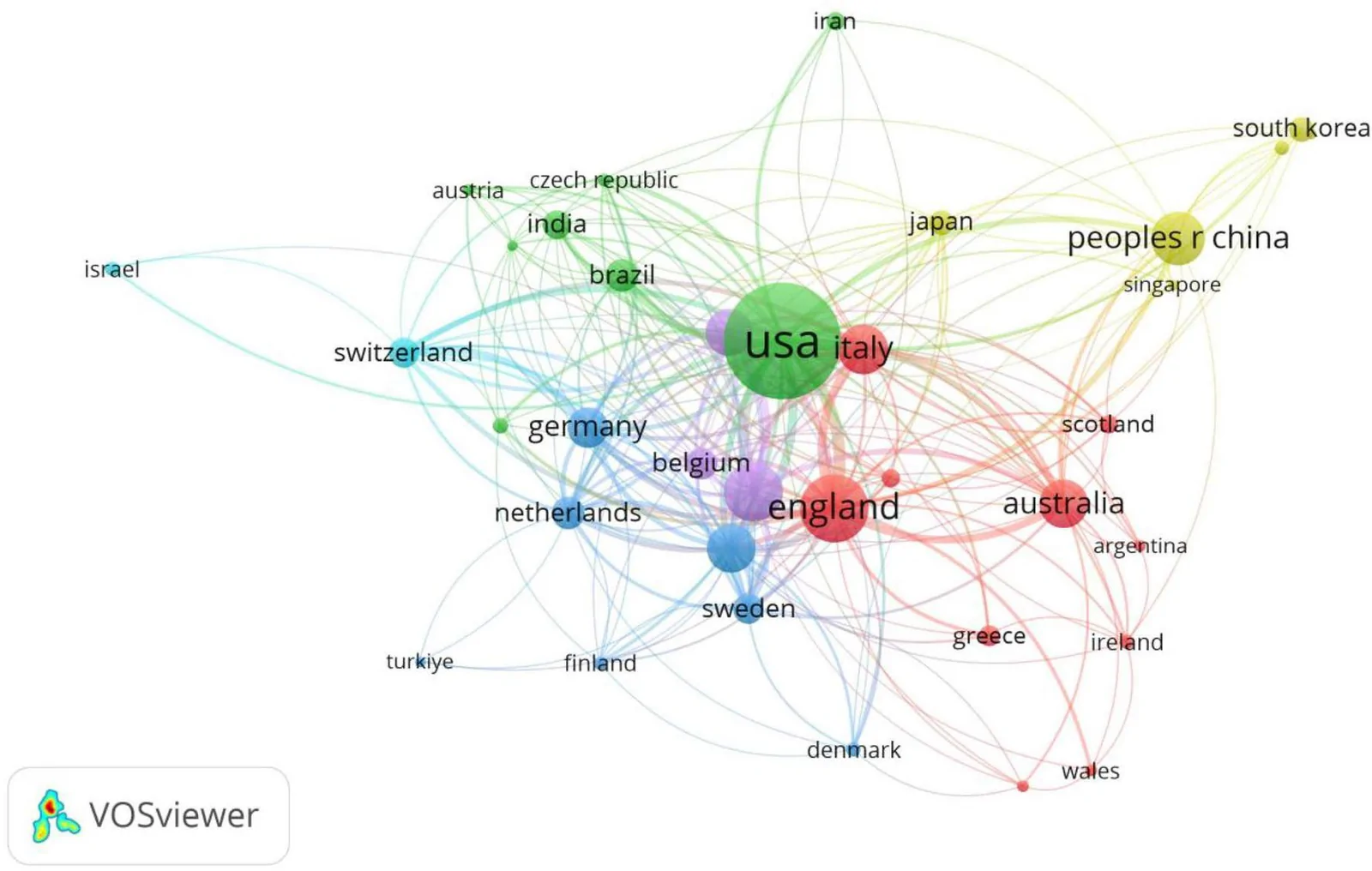
Country collaboration network (2001–2025).

The collected articles originated from 80 countries. This indicates that research on language comprehension in MCI is geographically widespread. However, the research is dominated by a small group of prolific countries. Figure 4 visualizes the co-authorship between productive countries. As can be seen from Figure 4, the United States is the most prolific country with the largest node in size, contributing 292 articles in the data set. England ranks second with 117 papers, and Canada (94), China (79), Italy (71), Spain (67), Australia (66) follow. France (64), Germany (50), and the Netherlands (34) complete the top ten list of the most productive countries in this research field.

Cooperative efforts are likely to center around the most productive regions in publications and tend to reflect geographic closeness (Zheng et al., 2016). As demonstrated in Figure 4, the USA shows thick collaborative links with several major contributors, including England, the Netherlands, Germany, Italy, Australia, and China. Dense co-authorship links are also obvious among European countries, especially among England, the Netherlands, Sweden, Germany, Switzerland, Belgium, and Italy, indicating active regional collaboration. In addition, China appears to form a relatively different collaboration cluster with Japan, Singapore, and South Korea, while still maintaining strong links with the USA and several Western countries. The color distribution further reflects that the country network can be divided into several collaboration clusters. One cluster is centered with the USA, another is organized around England and Australia, a third one includes the Netherlands, Sweden, Denmark, and nearby European countries, and the last cluster is associated with China and several East Asian countries.

Overall, the geographical distribution map indicates that research on language comprehension in MCI has developed into an international field, but this field is still dominated by several regional collaboration clusters, with the USA working as the center linking different groups.

### 3.3 Reference co-citation

**FIGURE 5:**
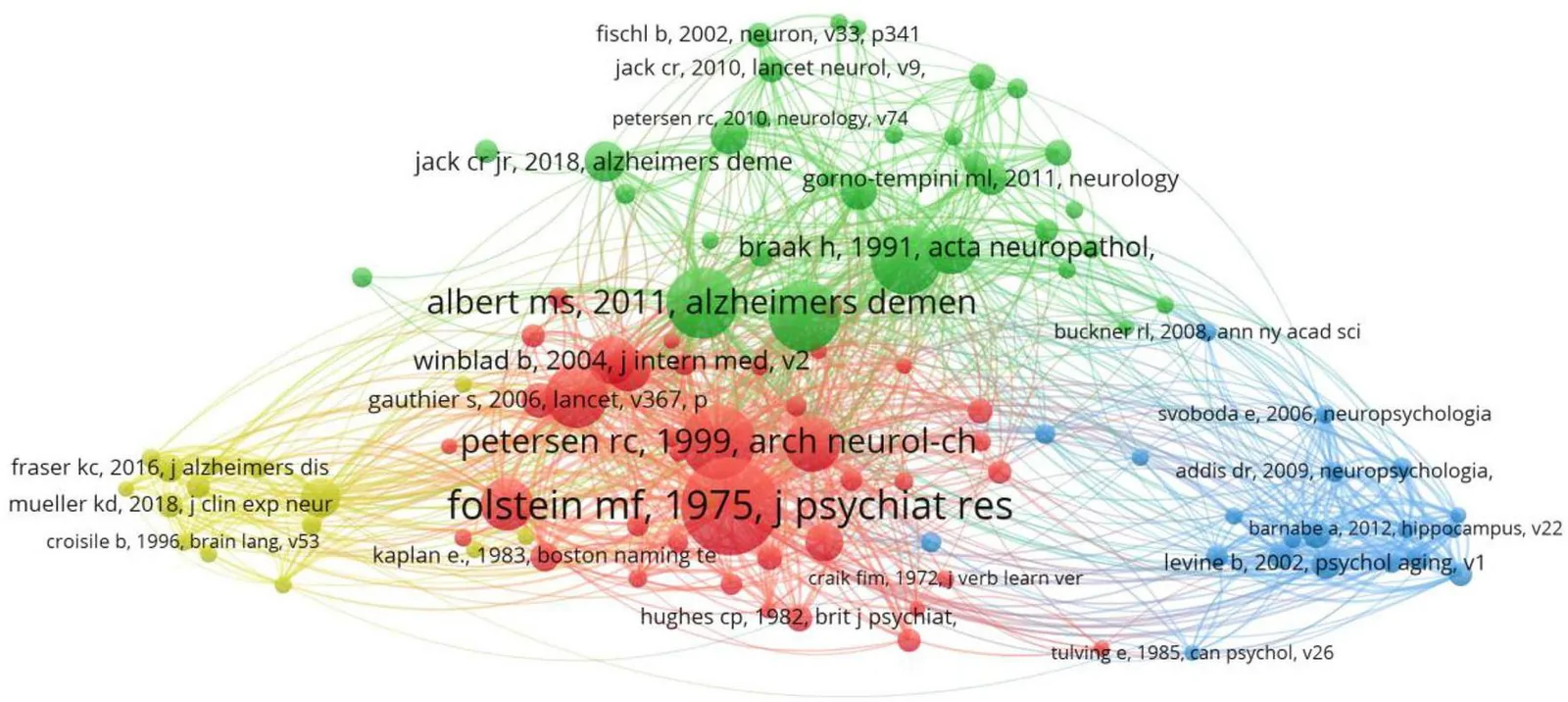
Reference co-citation network (2001–2025).

The co-cited references of the 914 records from 2001 to 2025 were visualized using the VOSviewer (Figure 5). In this map, each node represents a cited reference, and each link indicates the co-citation relationship between two cited articles.

The reference co-citation map visualizes that the knowledge foundation of this field is concentrated around a small set of highly influential articles. The most prominent node is Folstein et al. (1975), which introduced the Mini-Mental State Examination. Its prominence suggests that brief cognitive screening remains a shared background reference in studies of MCI and related language difficulties, because language comprehension findings in this population are often interpreted alongside global cognitive status rather than in isolation. Another salient node is Petersen et al. (1999), which characterized MCI as a clinical condition marked by memory deficits beyond that expected for age and education, when criteria for dementia were still not met. This article has remained foundational for participant definition in later research. For language comprehension research, its importance lies in that it provides a widely accepted clinical framework for identifying early stage cases in which subtle comprehension changes may be examined. Another two frequently cited articles are Albert et al. (2011) and Jack et al. (2018). Albert et al. (2011) proposed the National Institute on Aging and Alzheimer’s Association (NIA-AA) recommendations for the diagnosis of MCI due to AD and described different levels of diagnostic certainty based on biomarker evidence. Jack et al. (2018) further advanced the field by proposing the NIA-AA research framework and a biological definition of AD depending on the amyloid, tau and neurodegeneration [AT(N)] system. Their high citation frequency suggests that research on language comprehension in MCI is increasingly interpreted within more formal diagnostic and biomarker-based frameworks, especially when language-related issues are discussed as an early behavioral indicator rather than only as a general symptom. The fifth most cited reference is Braak and Braak (1991). The article described the neuropathological staging of AD-related changes. Its frequent co-citation indicates that the field is also grounded in research on pathological progression. This is also relevant to language comprehension research because comprehension deficits in MCI are often discussed in relation to the broader progression of AD pathology, even when language-related issues themselves are behavioral.

These five most cited articles suggest that the shared knowledge base of the field is built mainly on cognitive screening, the clinical characterization of MCI, and the diagnostic and biological features of AD. They are not language-comprehension studies in a narrow sense. Nevertheless, they provide the clinical, diagnostic, and pathological context within which language comprehension findings in MCI could be described, interpreted, and compared across studies.

### 3.4 Keyword co-occurrence

**FIGURE 6:**
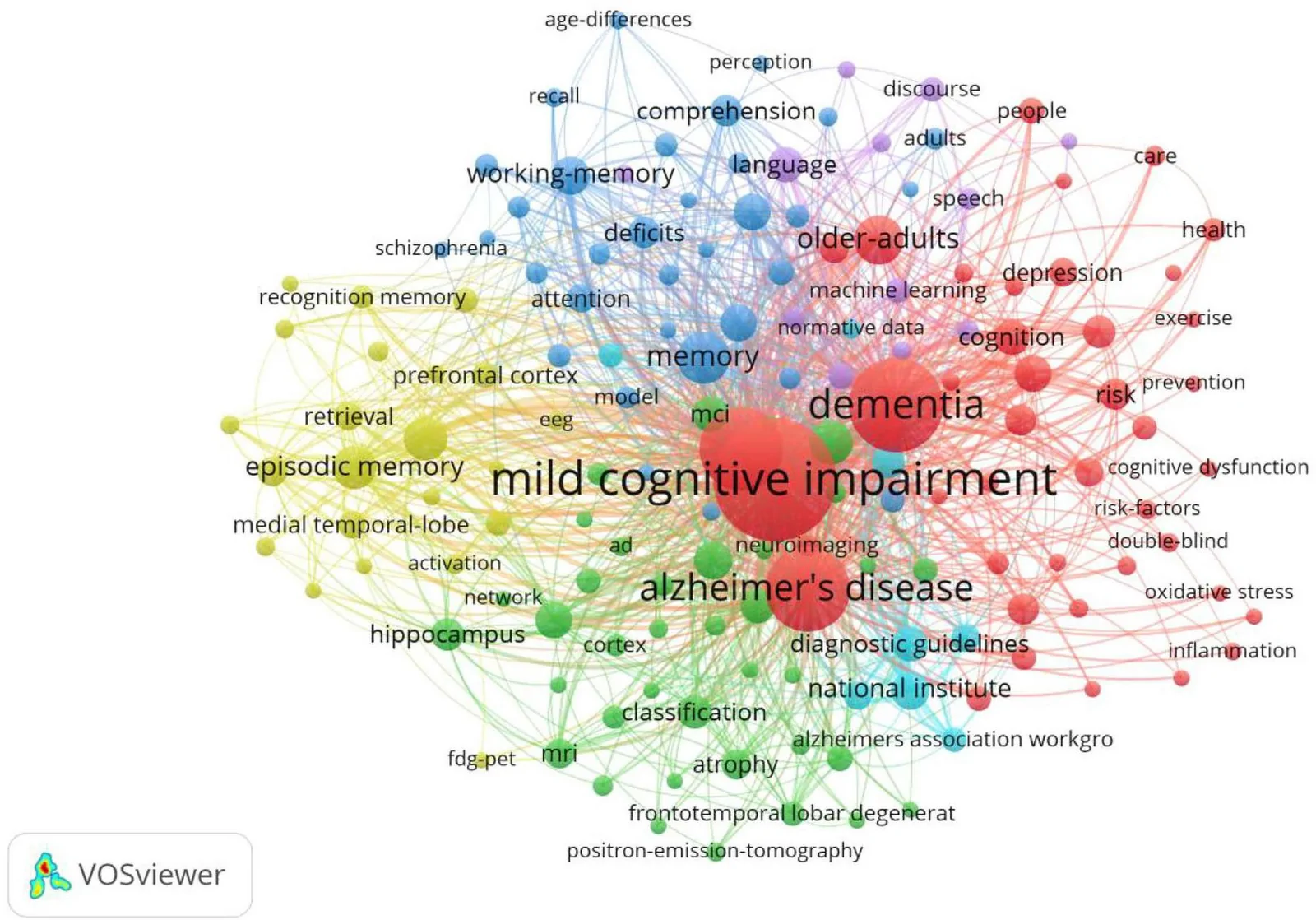
Keyword co-occurrence network (2001–2025).

The keyword co-occurrence network reflects that research on language comprehension in MCI is organized around several closely connected thematic areas. As shown in Figure 6, the largest and most central keywords include MCI, dementia, AD, memory, older adults, and episodic memory, indicating that the field is strongly grounded in cognitive aging, dementia-related conditions, and memory decline. These high-frequency keywords also suggest that language comprehension research in MCI is usually discussed in relation to the broader clinical and cognitive changes rather than as a completely independent topic.

According to Figure 6, several distinct keyword clusters can be identified. One large cluster is centered on MCI, dementia, cognition, risk, risk factors, depression, prevention, health, and care. This cluster reflects a clinical and public health perspective, in which MCI is studied together with dementia risk, cognitive dysfunction, prevention, and related health burdens. The prominence of these terms suggests that language-related research in MCI is often framed within broader discussions of diagnosis, intervention, and aging-related vulnerability. A second major cluster is associated with AD, classification, atrophy, MRI, FDG-PET, positron-emission tomography, neuroimaging, hippocampus, cortex, and frontotemporal lobar degeneration. This cluster indicates the biomarker and neuroimaging-related research. It suggests that studies on MCI and language-related deficits are frequently connected to structural and functional brain research, disease classification, and efforts to relate behavioral performance to underlying neural and pathological features. A third cluster is centered on memory, working memory, attention, comprehension, language, deficits, perception, recall, and age-differences. This cluster is more directly related to cognition and language processing. The co-occurrence of comprehension, and working memory suggests that language comprehension in MCI is often examined in relation to broader cognitive mechanisms, especially attentional control and memory-related resources. This pattern is consistent with the view that comprehension difficulties in MCI are not restricted to language alone, but are closely connected to declines in general cognitive support systems. Another noticeable cluster includes discourse, speech, adults, and older adults. This cluster points to a more language-centered line of research, especially at the discourse and spoken-language levels. The appearance of discourse and speech near highly connected terms such as language, comprehension, and older adults suggests growing interest in more naturalistic and ecologically relevant language measures in aging and MCI research. In addition, some keywords related to computational approaches, such as machine learning and normative data, are positioned near the center of the network. Although they do not form a large cluster, their location indicates that they are increasingly integrated into the field rather than remaining peripheral. This pattern suggests that recent research is beginning to combine traditional cognitive and clinical approaches with data-driven methods in detection, classification, and evaluation.

### 3.5 Reference co-citation clusters

**FIGURE 7:**
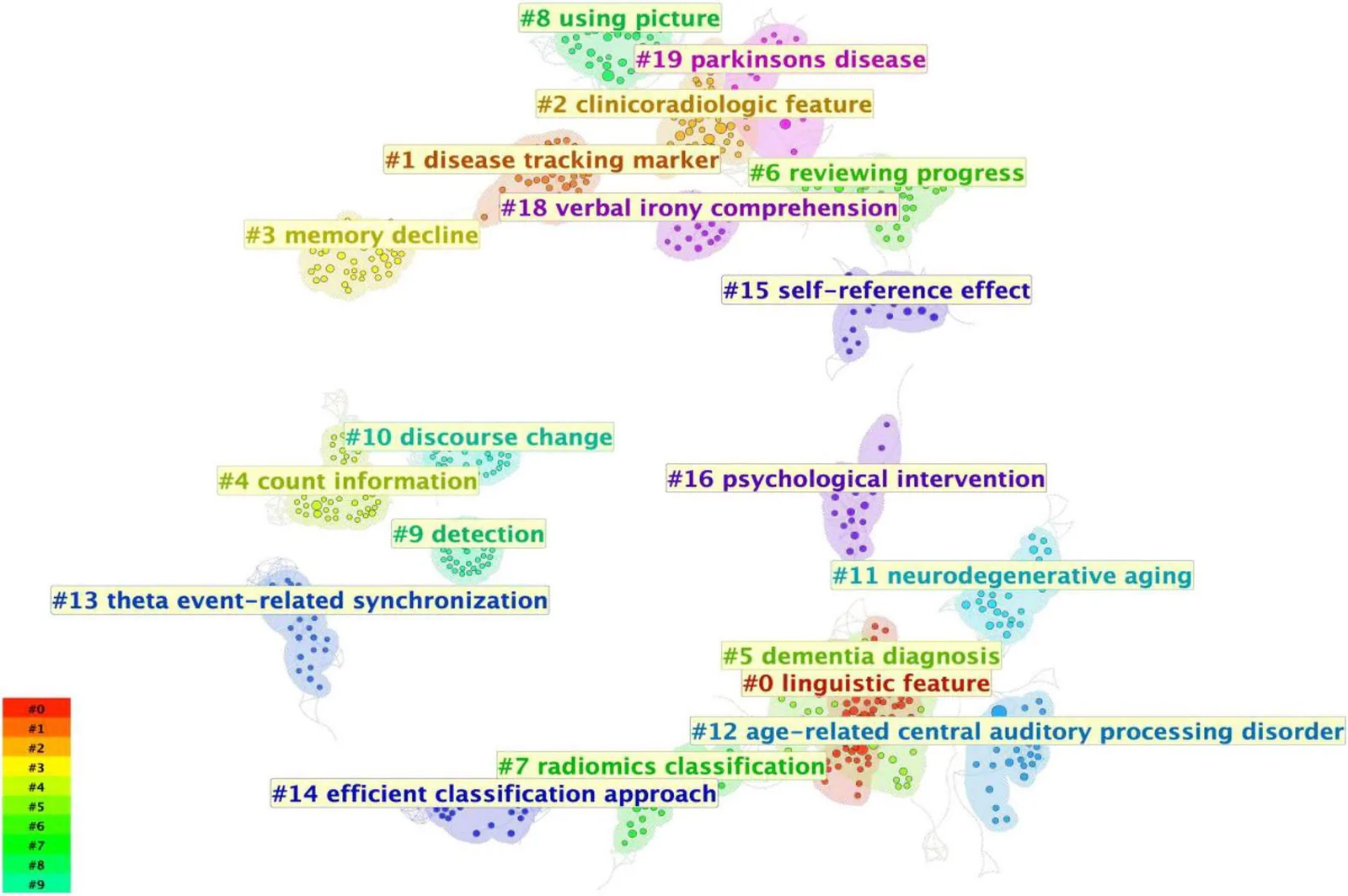
The co-citation clusters (2001–2025).

The reference co-citation network showed a high modularity Q of 0.9231 and a weighted mean silhouette value of 0.9691, indicating clear separation and strong internal consistency among the clusters. A total of 23 clusters were generated from the 914 articles, and all clusters showed silhouette values above 0.918, suggesting high cluster quality and a well-defined thematic structure. According to the narrative summary exported from CiteSpace, clusters #0 to #2 are the three largest clusters, indicating that these three clusters cover the main themes and research focuses during 2001–2025 in the research domain of language comprehension in MCI. The major clusters are illustrated in Figure 7.

Cluster #0 is labeled as linguistic feature. This cluster is mainly about speech and language changes across the AD continuum. The 5 most cited articles in cluster #0 are Alzheimer’s Association (2020), Mueller et al. (2018), Alzheimer’s Association (2023), Filiou et al. (2020), and Pistono et al. (2019). In 2020 Alzheimer’s Disease Facts and Figures, AD is described as a continuum including the preclinical stage, MCI due to AD, and dementia due to AD. AD of different stages can result in “language problems, impaired communication, and new problems with words in speaking or writing” (Alzheimer’s Association, 2020). The 2023 Alzheimer’s Disease Facts and Figures further describe “the patient journey from awareness of cognitive changes to potential treatment” (Alzheimer’s Association, 2023). These two reports were frequently cited by related articles, because they can provide a broader clinical context and background information of definitions, staging classifications, and current situations of the AD continuum. Mueller et al. (2018) suggested that connected speech can be a potentially sensitive linguistic indicator for AD. They also noted that picture-description task relied less on episodic memory and more on semantic knowledge and lexical retrieval. In this article, they mentioned a series of linguistic dimensions, such as semantic content, idea density, nonspecific language, grammatical complexity, coherence, and informativeness, in the language of individuals with AD. They described the language of adults with AD as “empty speech with circumlocutions, nonspecific language, and an overabundance of words conveying limited ideas” (Mueller et al., 2018). Filiou et al. (2020) reached a similar conclusion from a broader methodological perspective. Their scoping review states that connected speech deterioration appears early in both AD and MCI. Connected speech assessment may provide a quick, clinical speech tool for the early detection of subtle speech changes. Among the articles they reviewed, the most common outcomes were semantic and fluency outcome measures, while recurrent impairments included “anomia, grammatical complexity, idea density, discourse coherence, longer hesitations, a lower speech rate, and deterioration of semantic content and syntactic structure of speech” (Filiou et al., 2020). Pistono et al. (2019) investigated pauses in picture-based narrative (PBN) and memory-based narrative (MBN). Their results show that participants with AD produced more pauses in the PBN. They also found that pause frequency in the PBN was predicted by semantic fluency performance while pause frequency in the MBN was predicted by memory performance. Therefore, they argued that patients use pauses as a compensatory mechanism in the earliest stages of AD (Pistono et al., 2019). These frequently cited references suggest that Cluster #0 is generally organized around connected speech and related linguistic features in the AD continuum. Although these studies do not focus specifically on language comprehension in MCI, they provide an important background for MCI-related language comprehension research by emphasizing early language change, semantic processing, and discourse-level markers that may also be relevant to the study of comprehension.

Cluster #1, labeled disease tracking marker, mainly concerns the identification of markers for incipient AD and progression along the AD continuum. The most highly cited references in this cluster were Mattsson et al. (2009), Petersen and Morris (2005), Lonie et al. (2008), Palmer et al. (2008), and Jack et al. (2009). Mattsson et al. (2009) showed that cerebrospinal fluid (CSF) Aβ42, T-tau, and P-tau can identify incipient AD in patients with MCI with good accuracy, while also noting the need for standardization of analytical techniques and clinical procedures. Petersen and Morris (2005) framed MCI as a clinical entity and treatment target, and emphasized that careful clinical assessment together with neuropsychological testing can distinguish individuals likely to show cognitive deterioration from those with a benign prognosis. Lonie et al. (2008) examined clinical referral patterns and cognitive profiles in MCI, showing how case identification and neuropsychological profiles may vary across clinical settings. This reference further reflects the concern with MCI characterization and subtle cognitive differences in pre-dementia stages. Palmer et al. (2008) showed that MCI-multidomains had the highest progression to AD, and that the standard MCI criteria failed to identify some individuals with global cognitive deficits who nonetheless had a high risk of progressing to AD. Jack et al. (2009) used serial Pittsburgh compound B (PIB) and magnetic resonance imaging (MRI) to examine pathological progression across cognitively normal aging, MCI, and AD. Their findings suggested that neurodegeneration was more closely associated with cognitive decline than amyloid deposition alone, reflecting the complementary roles of MRI and PIB in tracking disease progression. Overall, Cluster #1 is organized around CSF biomarkers, MCI subtype definitions, predictive value for future AD, and serial imaging markers. Although these studies do not focus on language, they provide the clinical and pathological background for MCI language comprehension research, especially in relation to preclinical phases, multidomain impairment, and progression to AD.

Cluster #2 is labeled as clinicoradiologic feature. This cluster is mainly relevant to the diagnostic framework of the AD spectrum and the relation between clinical phenotype and biomarker evidence. The most highly cited references in this cluster were McKhann et al. (2011), Albert et al. (2011), Dubois et al. (2014), Gorno-Tempini et al. (2011), and Sperling et al. (2011). McKhann et al. (2011) defined AD dementia as the clinical syndrome arising from the AD pathophysiological process and noted that AD does not always present with memory impairment alone, but may also show nonamnestic presentations, including logopenic-primary progressive aphasia. Albert et al. (2011) extended this framework to MCI due to AD, the symptomatic predementia phase of AD, and stated that impairment may occur in memory, executive function, attention, language, and visuospatial skills. Dubois et al. (2014) further described AD as a clinicobiological entity, requiring an appropriate clinical AD phenotype together with a pathophysiological biomarker. In the same line, Gorno-Tempini et al. (2011) linked the logopenic variant of primary progressive aphasia to AD pathology and to *in vivo* biomarkers suggestive of AD, including PET-PIB positivity and decreased Aβ42 and increased tau in the CSF. Sperling et al. (2011) pushed the framework further back to preclinical AD, distinguishing AD-P from AD-C and describing early stages such as asymptomatic cerebral amyloidosis and amyloid positivity + evidence of synaptic dysfunction and/or early neurodegeneration. Taken together, these references show that Cluster #2 is structured around the combination of clinical phenotype, language-related atypical presentation, and pathophysiological biomarkers across the AD continuum. For the study of language comprehension in MCI, this cluster is relevant because it places language within the diagnostic description of MCI due to AD and also connects language-related phenotypes to the broader AD pathophysiological process.

Some clusters related to language are also worth noticing. Cluster #10 is labeled as discourse change, but its most cited references point more broadly to the interface between MCI characterization and discourse-level comprehension. Petersen et al. (1999) defined MCI as memory impairment beyond that expected for age and education without dementia, and Zwaan and Radvansky (1998) reviewed situation models in language comprehension and memory, that is, how readers build coherent mental representations during discourse processing. Read together, these references suggest that this cluster is concerned less with isolated language items than with discourse-level understanding built on clinically defined early cognitive change. Cluster #12 is labeled as age-related central auditory processing disorder. Its major references move the discussion toward hearing and cognition: Livingston et al. (2020) identified hearing loss as an important potentially modifiable risk factor for dementia, and Edwards et al. (2017) directly compared older adults with and without probable MCI on auditory processing tasks. This makes Cluster #12 relevant to language comprehension because spoken understanding depends not only on linguistic analysis, but also on auditory processing conditions that may already be altered in MCI. Cluster #18 is labeled as verbal irony comprehension, but its most cited references indicate a broader emphasis on semantic and language performance in early disease rather than irony alone. Joubert et al. (2010) showed that amnestic MCI patients present with naming deficits and genuine semantic impairment, Taler and Phillips (2008) reviewed language performance in AD and MCI, and Gainotti et al. (2014) focused on neuropsychological predictors of conversion from MCI to Alzheimer’s disease. These smaller clusters show that language-related work in this field extends beyond connected speech into discourse representation, auditory constraints on comprehension, and semantic vulnerability. For MCI language comprehension research, that matters. Comprehension difficulty is unlikely to reflect one isolated deficit. It is more likely shaped by several interacting levels, including discourse updating, auditory processing burden, and semantic change.

### 3.6 Citation bursts

Citation burst analysis identifies references that receive a rapid increase in citations during a particular period and is commonly used to trace changes in research attention (Chen, 2006; Wang et al., 2019). Recent or ongoing citation bursts may indicate topics that are receiving increasing scholarly interest. In the present study, particular attention was given to references whose bursts remained active through 2025 in order to identify the most recent research fronts.

**FIGURE 8:**
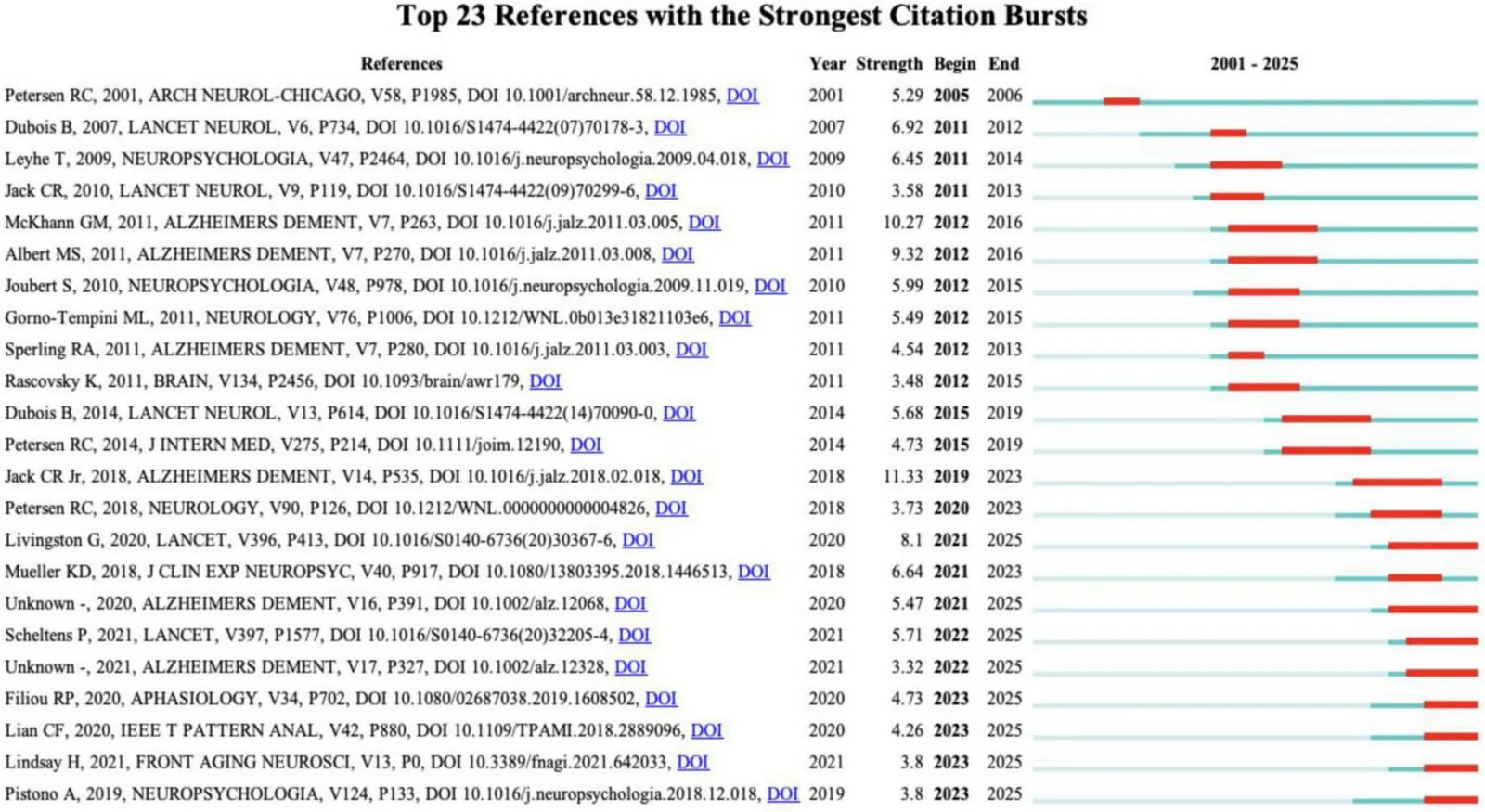
Top 23 references with the strongest citation bursts (2001–2025).

The citation burst analysis showed a comparatively clear development of research focus in this field, as illustrated in Figure 8. Early burst references were concentrated on the clinical concept of MCI, the diagnostic criteria of AD, and related neuropsychological characterization, including Petersen et al. (2001), Dubois et al. (2007), Leyhe et al. (2009), Jack et al. (2010), McKhann et al. (2011), Albert et al. (2011), Joubert et al. (2010), Gorno-Tempini et al. (2011), Sperling et al. (2011), Rascovsky et al. (2011), Dubois et al. (2014), and Petersen et al. (2014). Later citation bursts shifted further toward recent diagnostic frameworks and prevention, including Jack et al. (2018) and Petersen et al. (2018).

Among the references with citation bursts lasting to 2025, several studies provided broad frameworks for the field. Livingston et al. (2020) updated the prevention model and included hearing impairment among the modifiable risk factors, while also noting that amyloid-β and tau biomarkers indicate risk of progression but do not support pre-symptomatic diagnosis in everyday practice. The 2020 and 2021 Alzheimer’s disease facts and figures remained active burst references because they summarized the AD continuum, disease burden, and the expanding public health significance of dementia care. Scheltens et al. (2021) reinforced this direction by summarizing major developments in AD research, including the biological definition based on Aβ and tau biomarkers and the recognition of a language variant of AD.

Some articles with strong citation bursts up to 2025 were more directly related to language. Filiou et al. (2020) reviewed connected speech assessment in AD and MCI and showed that connected speech deterioration appears early, with semantic and fluency outcome measures being the most common and most relevant indicators in pathological aging. Lindsay et al. (2021) extended this research field with multilingual machine learning and identified AD-related deterioration of spontaneous speech that generalized across languages, with a primary impairment in semantics and mild syntactic impairment. Pistono et al. (2019) focused on pauses in early AD and found that pause frequency was related to semantic fluency performance and memory performance, suggesting compensatory mechanisms rather than simple breakdown. Lian et al. (2020) reflected the growing use of AI-based structural MRI approaches for AD diagnosis and neurodegenerative classification tasks through discriminative atrophy localization.

In short, the results of citation bursts show a shift from early work on clinical definition and diagnostic criteria to more recent attention to prevention, language-related measures, and automated detection. The references whose citation bursts continued through 2025 therefore mark the most active fronts at the end of the study period. For research on language comprehension in MCI, this pattern is informative. It suggests that comprehension-related findings are increasingly likely to be interpreted within a broader framework of early detection, disease staging, and language-related markers, rather than as isolated behavioral effects.

### 3.7 Cross-database validation based on Scopus

To examine the robustness of the WoS-based findings, an additional validation analysis was conducted using the Scopus database. The Scopus analysis combined descriptive cross-database validation with a formal comparison of the annual publication trends.

**FIGURE 9:**
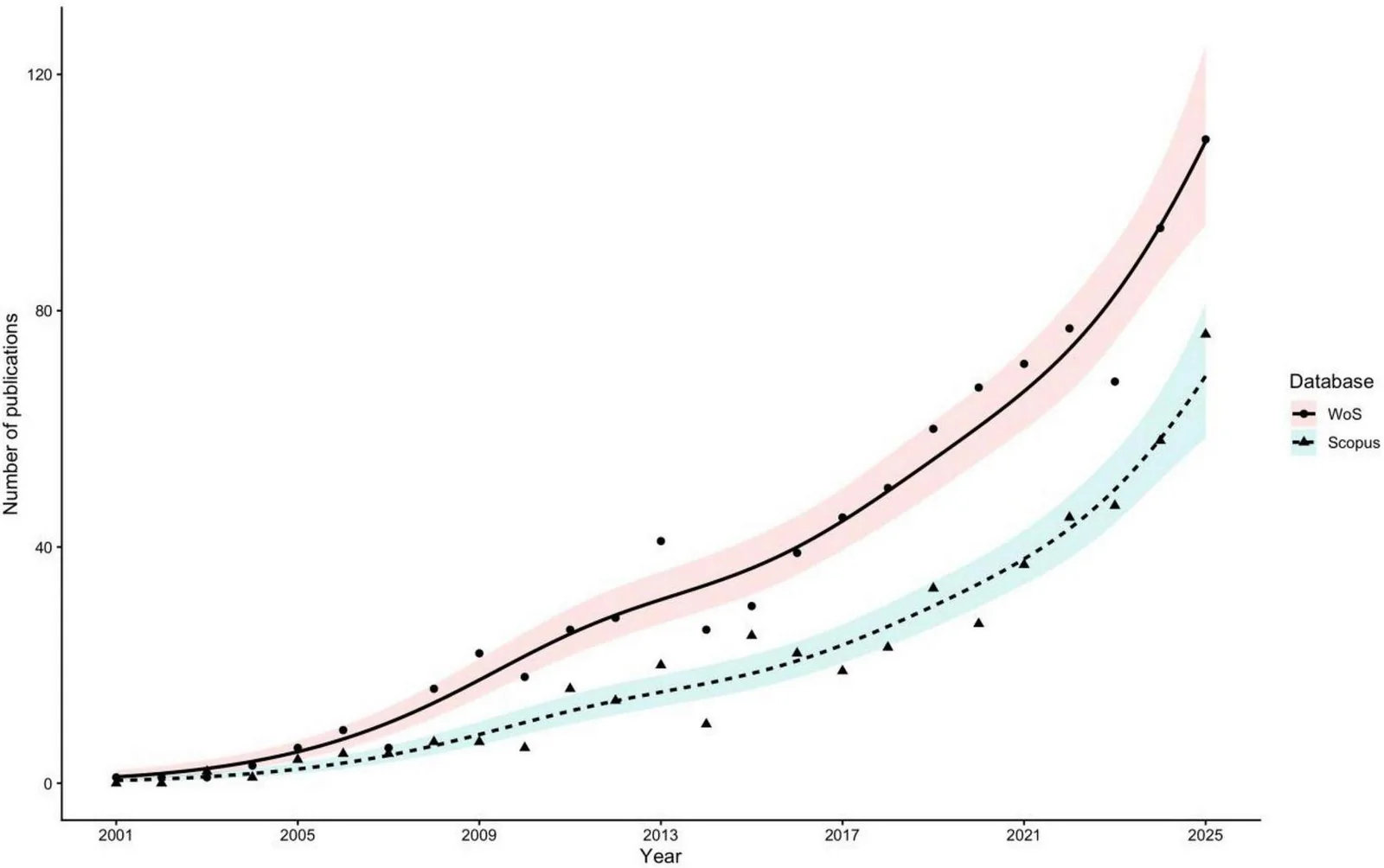
Generalized additive model estimates of annual publication trends in WoS and Scopus (2001–2025).

The Scopus dataset contained 509 publications between 2001 and 2025. Annual publication output was low during the early years but increased substantially in the later part of the study period, reaching its highest value in 2025. This descriptive pattern was broadly similar to that observed in the WoS dataset. To evaluate this similarity formally, a Poisson GAM was fitted to the annual publication counts from the two databases. The model showed a significant smooth effect of publication year, edf = 4.65, χ^2^ = 412.75, *p* < 0.001, with the effective degrees of freedom indicating a nonlinear temporal pattern. The difference smooth for Scopus relative to WoS was not statistically significant, edf = 1.30, χ^2^ = 1.81, *p* = 0.186, suggesting that the two annual publication trajectories did not differ significantly in shape. The parametric database effect was significant, *b* = -0.682, SE = 0.090, *z* = -7.62, *p* < 0.001. After adjustment for the temporal smooth, the estimated overall Scopus-to-WoS publication rate ratio was 0.51, 95% CI [0.42, 0.60]. The model explained 97.1% of the deviance in the observed annual counts. Negative-binomial and alternative basis-dimension models produced the same substantive conclusion; full results are provided in the Supplementary Materials. Figure 9 illustrates the higher overall publication volume in WoS and the broadly similar nonlinear shapes of the two fitted trajectories.

Beyond the annual publication trajectory, the productive journals identified in Scopus were also generally consistent with the WoS results. Journals related to Alzheimer’s disease, neuropsychology, neurology, neuroscience, and aging also occupied dominant positions. *Journal of Alzheimer’s Disease* showed the highest publication volume, followed by journals such as *Brain*, *Cortex*, *Journal of the International Neuropsychological Society*, *Neuropsychology*, and *Alzheimer’s and Dementia*. At the same time, language-related journals, like *Journal of Neurolinguistics*, also appeared among the top 15 leading journals (Table 2). This pattern further reflects the interdisciplinary nature of language comprehension research in MCI, which is closely connected with clinical neurology, cognitive neuroscience, neuropsychology, and language studies.

**TABLE 2:** The 15 most productive journals based on Scopus (2001–2025).

Rank	Title of journals	Number of published articles
1	Journal of Alzheimer’s Disease	19
2	Brain	8
3	Cortex	8
4	Journal of the International Neuropsychological Society	8
5	Medical Image Analysis	7
6	Neuropsychology	7
7	Alzheimer’s and Dementia	6
8	Annals of Neurology	6
9	BMJ Open	6
10	Brain Communications	6
11	Current Alzheimer Research	6
12	Frontiers in Neuroscience	6
13	Journal of Neurolinguistics	6
14	Frontiers in Aging Neuroscience	6
15	Neuroimage	6

**FIGURE 10:**
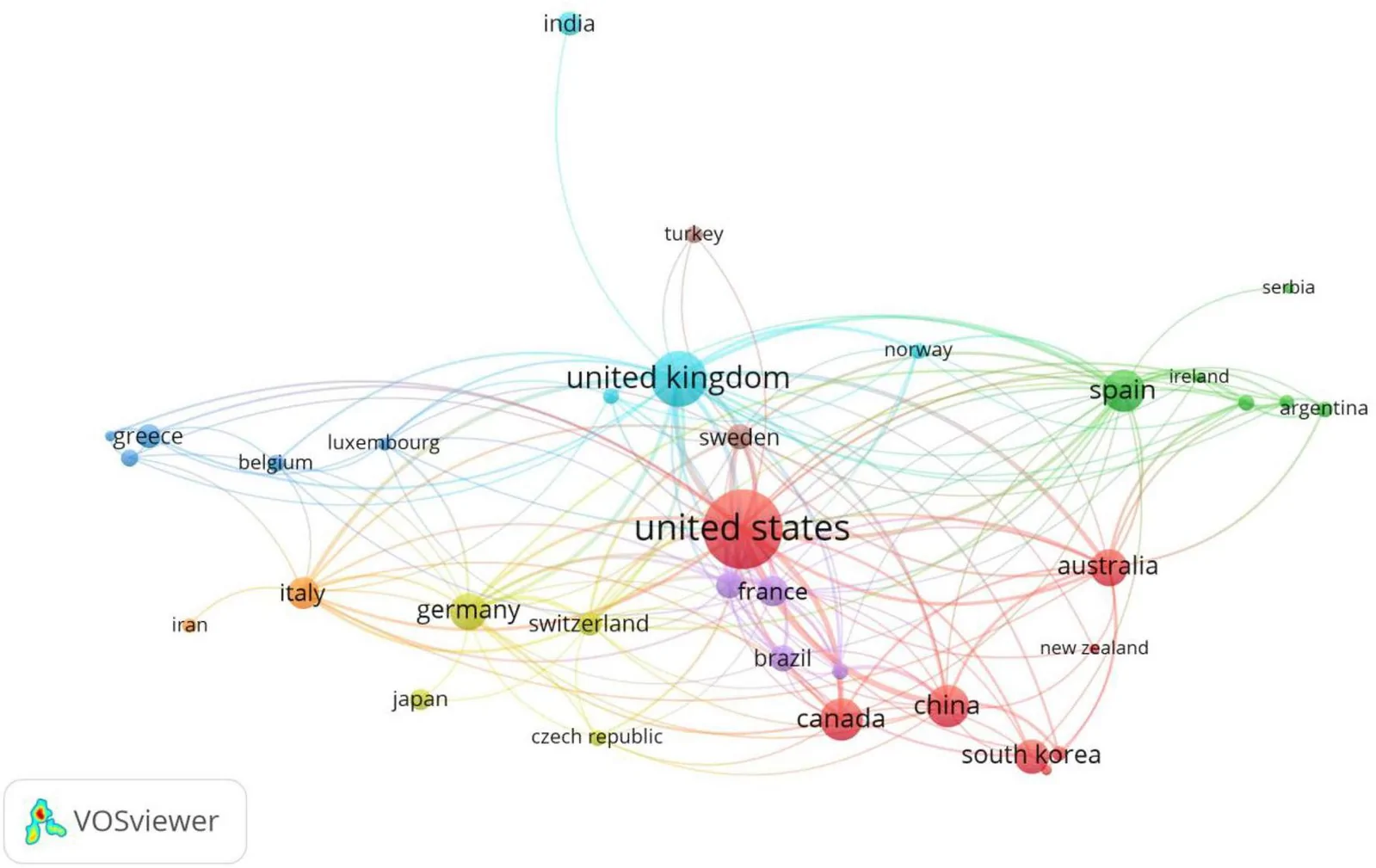
Country collaboration network based on Scopus (2001–2025).

The country collaboration network in Scopus showed a structure generally comparable to that identified in WoS. As is shown in Figure 10, the United States remained the most central and collaborative country in the network, with strong links to the United Kingdom, Spain, Australia, China, Canada, and several European countries. Similar regional collaboration clusters were observed across North America, Europe, and the Asia-Pacific region. These findings further support the dominant role of a limited number of countries in shaping research on language comprehension in MCI and related neurodegenerative conditions.

**FIGURE 11:**
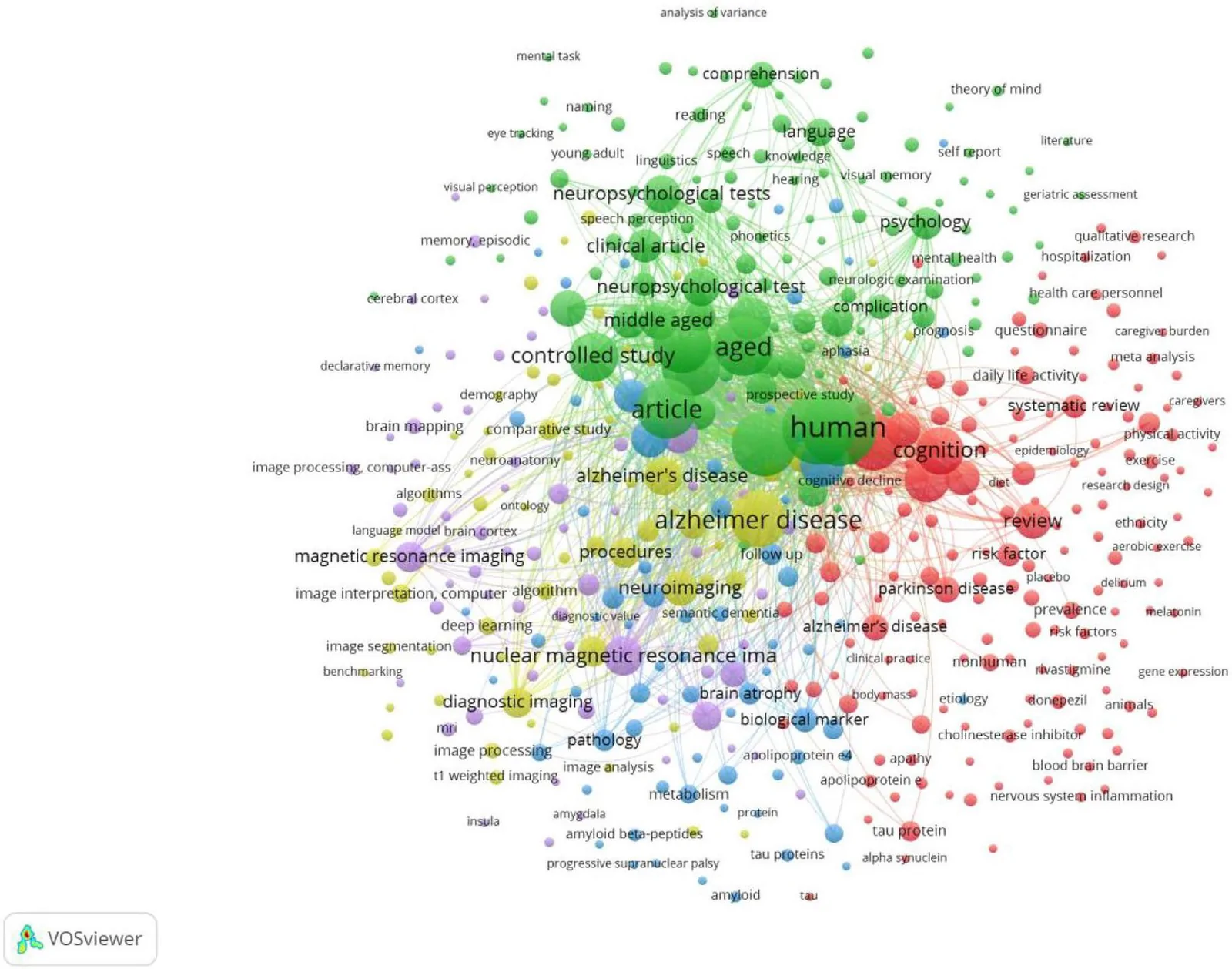
Keywords co-occurrence network based on Scopus (2001–2025).

Finally, the keyword co-occurrence analysis in Scopus also showed broad thematic convergence with the WoS findings. Important keywords such as Alzheimer’s disease, cognition, language, comprehension, neuropsychological test, and neuroimaging occupied central positions in the network (Figure 11). In addition, terms related to biomarkers, magnetic resonance imaging, semantic processing, and aging were closely connected with language-related topics. Compared with the WoS-based network, the Scopus keyword map also showed a relatively more explicit presence of language-related terms, such as speech perception, phonetics, and aphasia. Overall, these findings further support the view that current research on language comprehension in MCI is strongly embedded within broader frameworks of cognitive decline, AD-related pathology, neuropsychological assessment, and early detection research.

To make a brief summary, despite differences in database coverage, the Scopus results broadly reproduced the main WoS-based patterns in annual publication growth, leading journals and countries, and thematic keyword structure. This cross-database convergence supports the stability of the principal findings, although it does not indicate complete equivalence between the two databases.

## 4 Discussion

In relation to RQ1, the present study suggests that research on language comprehension in MCI has become increasingly active within the broader study of cognitive changes in neurodegenerative diseases. This pattern was evident in both databases. Although WoS contained substantially more records than Scopus, the GAM revealed no statistically significant difference in the shape of their annual publication trajectories across the two databases. The convergence of the two temporal patterns strengthens the interpretation that the observed increase reflects a broader development of the field rather than a pattern produced solely by the coverage of one database. This development should not be understood only in terms of publication growth. More importantly, it reflects a shift in researchers’ focus when they try to identify the pre-dementia stages of AD. Earlier MCI research was mainly concentrated on memory decline and the risk of progression to dementia (Petersen et al., 1999; Petersen and Morris, 2005). In comparison, the growing attention to language comprehension suggests that subtle cognitive changes are now being investigated through behaviors that are closer to everyday communication. Comprehension is not a simple language skill. It depends on attention, working memory, semantic access, inferencing, and contextual integration, so it can reveal decline that may not yet be obvious in general assessments. From this perspective, impairment in language comprehension should be understood as an integrative dimension of cognitive change, rather than a domain-specific deficit. However, the disciplinary distribution also suggests that this topic has not grown as an independent branch of linguistic research. The core journals publishing relevant papers are still dominated by AD research, neuropsychology, clinical neurology, and aging-related journals. Linguistics is present, but it is not yet at the center. This means that the field has gained research attention mainly because language-related findings are useful for clinical interpretation, not because comprehension itself has become a major theoretical focus. That situation indicates two sides. On the one hand, it keeps the topic closely connected to real clinical studies. On the other hand, it may also limit the depth of language-based explanation. The collaboration pattern suggests a similar conclusion. Research co-authorship is international, but it is still centered with a limited number of countries and regional clusters, with the USA occupying the most central position. This is understandable, because studies on MCI often depend on clinical cohorts, neuropsychological testing systems, imaging resources, and long-term interdisciplinary cooperation. However, for language comprehension research, such concentration raises an additional problem. Language-related findings are more sensitive than biological indicators to language structure, discourse conventions, educational experience, and communicative habits. Thus, more cross-linguistic and cross-cultural collaborations are needed, because it is necessary for the field to identify markers that are not too dependent on a few dominant research settings (Zheng et al., 2016). Similar publication trends, disciplinary distributions, and international collaboration patterns were also observed in the validation analysis based on Scopus, suggesting that these macro-level structures were relatively stable across databases.

Regarding RQ2, the findings suggest that the field has developed within a strongly clinical and AD-related knowledge structure. The most influential references are not specific studies on language comprehension, but studies that define MCI, describe AD pathology, and establish diagnostic and biomarker frameworks, such as Folstein et al. (1975), Braak and Braak (1991), Petersen et al. (1999), Albert et al. (2011), and Jack et al. (2018). This pattern suggests that language comprehension research in MCI has usually been interpreted through questions of clinical definition, disease staging, and progression risk, rather than through theories of comprehension itself. Such a structure gives the field a certain stability, given that language-related findings can be discussed within recognized frameworks of MCI and AD. But it also has a limitation. When the shared knowledge foundation is structured by disease models, language may easily be treated as one more behavioral signal, while the internal structure of comprehension receives less attention. This may explain why many studies agree that language changes matter in MCI, but they are less precise about which part of comprehension is changing and why the part is changing. Similar thematic associations were also identified in the Scopus keywords co-occurrence, in which language-related terms remained closely connected with cognition, neuropsychological assessment, neuroimaging, and AD-related studies. The thematic pattern identified in this study reflects a more complex explanation. The recurrent association of comprehension with working memory, attention, discourse, speech, and semantic processing suggests that researchers are not dealing with one isolated deficit. Rather, comprehension seems to become fragile when tasks place heavier demands on integration across time, context, and levels of representation. Earlier empirical studies support this interpretation. Difficulties often become more obvious when understanding depends on inference, discourse-level integration, metaphor processing, irony interpretation, or the maintenance of a coherent situation model, rather than only lexical access or simple sentence decoding (Taler and Phillips, 2008; Cardoso et al., 2014; Gaudreau et al., 2013; Drummond et al., 2015; Maziero et al., 2021; Yang and Huang, 2023). Recent studies on SCD also move in the same direction, showing that sentence processing becomes more vulnerable when structural demands increase, and that working memory may shape the relation between cognitive reserve and comprehension performance (López-Higes et al., 2024, 2025). The cluster analysis strengthens this view. On the one hand, references on connected speech and linguistic features, such as Mueller et al. (2018), Filiou et al. (2020), and Pistono et al. (2019), emphasize early changes in discourse, semantic content, fluency, and pause behavior. On the other hand, co-citation clusters about disease tracking and clinicoradiologic features, including Mattsson et al. (2009), McKhann et al. (2011), Dubois et al. (2014), and Sperling et al. (2011), indicate that these language-related phenomena are important when they can be connected to the AD continuum and to the prediction of cognitive decline. Smaller clusters add further interpretations. Research related to discourse models suggests that comprehension difficulty may involve problems in building and updating situation models. Clusters organized around auditory processing and verbal irony imply that spoken comprehension, semantic vulnerability, and higher-level pragmatic interpretation may all be relevant to early cognitive change (Joubert et al., 2010; Edwards et al., 2017; Livingston et al., 2020). In brief, language comprehension in MCI is unlikely to be studied in one single way. It is more likely to be structured by several interacting domains. This also helps to explain why the literature has not always been fully consistent. As Kokje et al. (2022) argued, different tasks, materials, and scoring methods can lead to different findings. The inconsistency should not always be treated as a weakness. It reflects the multidimensional nature of comprehension itself to some extent.

With respect to RQ3, the burst analysis suggests that the field is entering a new stage characterized by prevention-oriented research, ecologically valid assessment, and computational approaches. Earlier influential research mainly focused on defining MCI, refining diagnostic criteria, and describing the clinical profile of early impairment, as seen in Petersen et al. (2001), Dubois et al. (2007), Leyhe et al. (2009), Albert et al. (2011), Gorno-Tempini et al. (2011), and Petersen et al. (2014). More recent burst references demonstrate a shift toward prevention, ecologically relevant language assessments, and data-driven detection, with Jack et al. (2018), Petersen et al. (2018), Livingston et al. (2020), Scheltens et al. (2021), and the Alzheimer’s Association (2020, 2021) providing the broader context. This shift implies that language-related findings are increasingly valued not only as descriptive features, but also as potential early markers which are relevant to clinical and public-health settings. Studies on connected speech are particularly meaningful here. Although connected speech is not equivalent to comprehension itself, successful connected speech production and discourse organization rely heavily on semantic integration, contextual maintenance, and inferential processes that overlap substantially with comprehension. These communicative processes are closely associated with successful understanding in everyday communication. Reviews by Mueller et al. (2018) and Filiou et al. (2020), together with the narrative findings of Pistono et al. (2019), suggest that speech and discourse measures may capture subtle change in forms of communication that are closer to daily life than many traditional tasks. That direction is reasonable and well grounded. Many real-life comprehension problems do not first appear in isolated word recognition or simple sentence judgment. They are more likely to appear when older adults need to follow extended discourse, integrate implied meaning, understand non-literal expressions, or process spoken language under heavier cognitive load. Future research should therefore pay more attention to ecologically valid comprehension tasks, especially tasks that involve discourse updating, inference generation, pragmatic interpretation, and auditory-supported understanding. However, this does not mean that every naturalistic task is automatically better than the one conducted in labs. More ecological tasks are often harder to score, less controlled, and more easily influenced by education, hearing status, and cultural background. So the field needs a better balance between ecological validity and precision of assessment. Another research direction is the growing role of computational methods. Studies such as Lindsay et al. (2021) and Lian et al. (2020) indicate that machine learning, multilingual speech analysis, and multimodal prediction have become increasingly visible in this field. This situation is promising, but it also requires more caution. A model may successfully classify individuals while still leaving unclear what aspect of comprehension it is actually detecting. Is it capturing semantic degradation, discourse disorganization, reduced inferential efficiency, auditory difficulty, or a broader decline in cognitive control? These questions cannot be solved by accuracy alone. For this reason, future studies should build closer relationships between computational indicators and theoretically defined components of comprehension. It may also be useful to compare different levels of language processing more systematically. For example, sentence-level versus discourse-level comprehension, literal versus non-literal understanding, and visual versus auditory presentation. Such comparisons can help clarify which aspects are most sensitive in early stages, and which are more strongly affected by general cognitive burden. In addition, more longitudinal research is needed. Cross-sectional findings can suggest association, but they cannot show clearly whether a given comprehension assessment has real predictive value for progression from MCI to AD or other dementias.

The present findings further suggest that language comprehension in MCI may be better conceptualized as an interactional and resource-dependent cognitive process rather than as a purely linguistic function. Comprehension difficulties may become particularly apparent when successful understanding requires the continuous coordination of semantic, discourse, inferential, attentional, and executive resources. This perspective provides a possible explanation for why findings across previous studies have varied according to task type, modality, and processing demands. Overall, the present study indicates that research on language comprehension in MCI has entered a more active and diversified stage, while also highlighting that comprehension should be considered within the broader domain of cognitive and communicative changes associated with neurodegenerative diseases. Although the present review focused specifically on comprehension, language production and other expressive abilities may also show subtle changes in MCI. Future bibliometric and systematic reviews could extend this perspective by examining how research on receptive and expressive language abilities has developed across the MCI-AD continuum and how these abilities jointly contribute to everyday communication.

While the present findings provide a broad view of the development of this field, several limitations should be considered when interpreting the results. First, the analysis was based on English-language articles and reviews indexed in the WoS Core Collection. Although the Scopus validation supported the robustness of the major publication patterns and thematic structures, studies published in other languages, regional journals, or other publication formats may have been underrepresented. This limitation is particularly relevant for language comprehension research, as linguistic structures, discourse conventions, and assessment approaches vary across languages and cultures. Second, WoS and Scopus differ in journal coverage, indexing practices, metadata structures, and publication volume. Therefore, the convergence observed across databases should be interpreted as consistency in overall patterns rather than statistical equivalence between databases. Third, bibliometric findings are shaped not only by the literature itself but also by analytical decisions, including keyword selection, inclusion thresholds, counting methods, clustering procedures, and software settings. Citation and co-citation patterns further reflect scholarly visibility and intellectual connectivity rather than methodological quality or empirical strength. Accordingly, the maps presented here should be viewed as analytical representations of the retrieved literature rather than exhaustive descriptions of the field. Finally, bibliometric analysis provides a broad overview of research development but cannot replace detailed evaluation of individual studies, assessment instruments, or empirical effect sizes. Although transparent reporting can improve the reproducibility and interpretability of bibliometric research, such mapping does not constitute a comprehensive strategic evaluation framework on its own. Future studies may therefore combine bibliometric evidence with systematic reviews, meta-analyses, and complementary qualitative approaches to further evaluate research priorities and translational opportunities (c.f., Arsyi et al., 2026).

## 5 Conclusion

In the current study, a bibliometric analysis was conducted on literature concerning language comprehension in MCI published from 2001 to 2025, drawing on the WoS Core Collection and cross-validated by Scopus. Using CiteSpace and VOSviewer, this study quantitatively analyzed and visually mapped the retrieved literature. The analysis covered publication trends, major journals and countries, category distributions, co-citation patterns, keyword networks, thematic clusters, and citation bursts. The results suggest that language comprehension is receiving growing attention, for it can serve as an early indicator of neurodegeneration across the full MCI progression spectrum. The mapping further indicates that this field has developed mainly within the broader frameworks of MCI staging, the AD continuum, biomarker research, and behavioral indicators. Within these shared frameworks, language comprehension is treated not only as a clinical symptom, but also as a potentially sensitive sign of early cognitive change in neurodegenerative diseases. It is also analyzed in a more process-oriented way, especially in relation to discourse processing, semantic integration, inference, and general cognitive support systems. At the same time, recent studies reflect a growing interest in language tasks with high ecological validity, automated detection, and the use of language-related assessments in clinical and care settings. Admittedly, because this field is highly interdisciplinary and includes different task types, research traditions, and clinical perspectives, one single mapping cannot provide a fully complete overview.

Several directions deserve further attention in future research. First, more longitudinal and stage-sensitive studies are needed, so that SCD, MCI, and later AD-related stages can be examined as a developmental process rather than only as discrete groups. Second, more cross-linguistic and cross-cultural evidence is needed, because current findings still come mainly from a limited number of languages and research contexts. Third, future studies should make clearer distinctions across different aspects of comprehension, such as sentence-level, discourse-level, pragmatic, and auditory-supported comprehension, and connect these more directly with clinical features and biomarker information. This may help explain not only whether language comprehension changes, but also how and why it changes within the AD continuum. Fourth, as naturalistic tasks and computational methods receive more attention, the field also needs more reliable, repeatable, and theoretically clear measures. Only in this way can language-related findings be compared more confidently across studies and be used more carefully in follow-up, early detection, and intervention evaluation. In addition, future work could examine language production and other expressive abilities alongside comprehension in order to provide a more complete account of communicative change in MCI. These directions may help the field develop from merely describing comprehension difficulty to a clearer and deeper understanding of its mechanisms, timing, and practical value in MCI research.

## Data Availability

The original contributions presented in the study are included in the article/Supplementary material, further inquiries can be directed to the corresponding author.
