# Supplementary material for: Language comprehension in mild cognitive impairment: a bibliometric study of research trends and thematic structures
Source: Front Psychol. 2026 Aug 26;17:1886461. doi: 10.3389/fpsyg.2026.1886461 (PMC13553494; doi:10.3389/fpsyg.2026.1886461)
Supplement: Supplementary file 1 [file Table_1.docx]

**Supplementary Materials**

*Language comprehension in mild cognitive impairment: A bibliometric study of research trends and thematic structures* (Manuscript ID: 1886461)

# **Supplementary Table S1. Database information and complete search strategies**

| **Item** | **Web of Science Core Collection** | **Scopus validation dataset** |
| --- | --- | --- |
| **Platform and provider** | Web of Science Core Collection, accessed through the Web of Science platform provided by Clarivate | Scopus, provided by Elsevier |
| **Database coverage** | Science Citation Index Expanded (SCI-E); Social Sciences Citation Index (SSCI); Arts and Humanities Citation Index (A&HCI); Conference Proceedings Citation Index–Science (CPCI-S); Conference Proceedings Citation Index–Social Science and Humanities (CPCI-SSH) | Multidisciplinary coverage provided by the Scopus database |
| **Final retrieval date** | January 1, 2026 | March 12, 2026 |
| **Time span** | January 1, 2001-December 31, 2025 | January 1, 2001-December 31, 2025 |
| **Search field** | Topic (TS) | Article title, Abstract, Keywords (TITLE-ABS-KEY) |
| **Complete search string** | TS = ((“mild cognitive impairment” OR “mild neurocognitive disorder” OR MCI) AND (sentence* OR discourse OR text* OR narrative* OR pragmatic* OR inferen* OR metaphor* OR irony OR semantic*) AND (comprehension OR comprehend* OR understanding OR understand* OR processing OR process* OR interpret* OR integration)) | TITLE-ABS-KEY((“mild cognitive impairment” OR “mild neurocognitive disorder” OR MCI) AND (sentence* OR discourse OR text* OR narrative* OR pragmatic* OR inferen* OR metaphor* OR irony OR semantic*) AND (comprehension OR comprehend* OR understanding OR understand* OR processing OR process* OR interpret* OR integration)) |
| **Language restriction** | English | English |
| **Document types** | Articles and reviews (book reviews excluded) | Articles and reviews (book reviews excluded) |
| **Final records included** | 914 | 509 |
| **Purpose of dataset** | Primary bibliometric analysis | Independent cross-database validation; Scopus records were analyzed separately and were not merged with the WoS dataset. Descriptive comparisons were supplemented by a GAM comparison of annual publication trajectories. |

# **Supplementary Table S2. Data export information**

| **Database** | **Analytical purpose** | **Software** | **Export format** | **Record content** | **Export files** |
| --- | --- | --- | --- | --- | --- |
| **WoS Core Collection** | Primary CiteSpace analyses | CiteSpace 6.4.R1 | Plain text files (.txt) | Full record and cited references | Exported as three sequential files because of database export limits. |
| **WoS Core Collection** | Country co-authorship, cited-reference co-citation, and keyword co-occurrence analyses | VOSviewer 1.6.19 | Tab delimited files (.txt) | Full record and cited references | Exported as three sequential files because of database export limits. |
| **Scopus** | Cross-database validation: country collaboration and keyword co-occurrence | VOSviewer 1.6.19 | CSV (.csv) | Citation information; bibliographical information; abstracts and keywords; funding details; references; and other information | Exported as one file. |
| **WoS and Scopus** | Annual publication trajectory comparison and GAM analysis | Microsoft Excel for data preparation; R 4.6.1 with *mgcv* 1.9-4 for GAM analysis | CSV (.csv) | Publication year, database, and annual publication count | One combined analysis file |

# **Supplementary Table S3. VOSviewer settings and thresholds**

| **Dataset** | **Network** | **Analysis type** | **Unit of analysis** | **Counting method** | **Minimum threshold** | **Items meeting threshold** | **Normalization** |
| --- | --- | --- | --- | --- | --- | --- | --- |
| **WoS (n = 914)** | **Country collaboration** | Co-authorship | Countries | Full counting | Minimum of 5 documents per country | 37 countries | Association strength |
| **WoS (n = 914)** | **Reference co-citation** | Co-citation | Cited references | Full counting | Minimum of 15 citations per cited reference | 107 cited references | Association strength |
| **WoS (n = 914)** | **Keyword co-occurrence** | Co-occurrence | All keywords | Full counting | Minimum of 5 occurrences per keyword | 378 keywords | Association strength |
| **Scopus (n = 509)** | **Country collaboration** | Co-authorship | Countries | Full counting | Minimum of 5 documents per country | 29 countries | Association strength |
| **Scopus (n = 509)** | **Keyword co-occurrence** | Co-occurrence | All keywords | Full counting | Minimum of 5 occurrences per keyword | 481 keywords | Association strength |

# **Supplementary Table S4. CiteSpace settings, parameters, and network diagnostics**

| **Analysis** | **Parameter** | **Setting** |
| --- | --- | --- |
| **General** | Software version | CiteSpace 6.4.R1 |
|  | Time span | 2001-2025 |
|  | Years per slice | 1 year |
| **Node selection** | Selection criterion | g-index |
|  | g-index scale factor (k) | k = 25 |
|  | Link retaining factor (LRF) | 2.5 |
|  | Maximum links per node (L/N) | 10 |
|  | Look-back years (LBY) | 5 |
|  | Exponential parameter (e) | 1.0 |
| **Pruning** | Pathfinder | Yes |
|  | Pruning sliced networks | Yes |
|  | Minimum spanning tree | No |
|  | Pruning the merged network | No |
| **Subject category analysis** | Node type | Category |
|  | Network  Largest 1 CCs | Network: N = 115, E = 259 (Density = 0.0395)  Largest 1 CCs: 96 (83%) |
| **Reference co-citation clustering** | Node type | Reference |
|  | Labeling method | LLR |
|  | Number of reference co-citation clusters | 23 |
|  | Cluster silhouette values | All 23 clusters had silhouette values above 0.918 |
|  | Network  Largest 1 CCs  Modularity Q  Weighted Mean Silhouette S  Harmonic Mean (Q, S) | Network: N = 987, E = 2414 (Density = 0.005)  Largest 1 CCs: 734 (74%)  Modularity Q = 0.9231  Weighted Mean Silhouette S = 0.9691  Harmonic Mean (Q, S) = 0.9456 |
| **Citation burst analysis** | Node type | Reference |
|  | Burst-detection settings | Detection model:   Initial state ratio, : 2.0  Successive state ratio, : 2.0  Number of states: 2  Gamma, : 1.0  Minimum duration: 2 years |
|  | Output | Number of references identified with citation bursts: 23 |

# **Supplementary Table S5. Eligibility criteria for manual relevance screening**

| **Domain** | **Inclusion criteria** | **Exclusion criteria** |
| --- | --- | --- |
| **Overall topic relevance** | The record was substantively related to language comprehension in MCI. | MCI or language comprehension was mentioned only incidentally. |
| **Language-comprehension relevance** | The study addressed comprehension or closely related language-processing issues, such as sentence, discourse, semantic, inferential, pragmatic, narrative, or text-level processing. | The study focused on unrelated cognitive, neurological, or biological processing without a substantive language-comprehension component. |
| **Population or condition relevance** | MCI was a substantive population, diagnostic category, or clinical focus. | The record focused on other conditions without a clear MCI-related component. |
| **Screening basis** | Relevance was assessed from the title and abstract; full texts were consulted when necessary. | Records were excluded when relevance could not be established after title, abstract, and, where necessary, full-text review. |

*Note. Language and document type restrictions were applied through database refinement before manual relevance screening and were therefore not reassessed at this stage.*

# **Supplementary Table S6. Generalized additive model specification, results, sensitivity analyses, and diagnostics**

| **Section** | **Item** | **Specification or result** |
| --- | --- | --- |
| **Data** | Study period | 2001–2025 |
|  | Observations | 50 annual database-specific counts (25 years × 2 databases) |
|  | Records represented | WoS: 914; Scopus: 509 |
| **Primary model** | Software | R 4.6.1; mgcv 1.9-4 |
|  | Response variable | Annual publication count |
|  | Distribution and link | Poisson distribution with a log link |
|  | Model formula | publications ~ database + s(year_centered, k = 8) + s(year_centered, by = database_diff, k = 8) |
|  | Year transformation | year_centered = year − 2001 |
|  | Reference database | Web of Science |
|  | Spline and estimation | Penalized thin-plate regression spline; restricted maximum likelihood |
|  | Basis dimension | k = 8 |
| **Primary results** | Overall temporal smooth | edf = 4.651; Ref.df = 5.477; χ² = 412.751; p < .001 |
|  | Scopus-WoS difference smooth | edf = 1.298; Ref.df = 1.535; χ² = 1.813; p = .186 |
|  | Database effect | b = −0.68198; SE = 0.08953; z = −7.617; p < .001 |
|  | Rate ratio | Scopus/WoS = 0.506; 95% CI [0.424, 0.603] |
|  | Model fit | Adjusted R² = .971; deviance explained = 97.1%; AIC = 277.808 |
| **Alternative distribution** | Negative-binomial model | Estimates were virtually identical; theta = 2,300,299.668; AIC = 279.808 |
| **Sensitivity analysis** | k = 6 | Overall smooth: edf = 4.058, p < .001; difference smooth: edf = 1.325, p = .192; AIC = 277.473; deviance explained = 97.0% |
|  | k = 8 | Overall smooth: edf = 4.651, p < .001; difference smooth: edf = 1.298, p = .186; AIC = 277.808; deviance explained = 97.1% |
|  | k = 10 | Overall smooth: edf = 4.943, p < .001; difference smooth: edf = 1.291, p = .185; AIC = 278.019; deviance explained = 97.2% |
| **Diagnostics** | Convergence | Full convergence; positive-definite Hessian; full model rank |
|  | Basis-dimension check | k-index = 0.88; p = .12 for the overall smooth and p = .20 for the difference smooth; no evidence that k was too low |
|  | Residual plots | No marked systematic pattern; observed and fitted values showed close agreement |
|  | Residual autocorrelation | No persistent autocorrelation pattern was evident in the database-specific residual ACF plots |
| **Interpretation** | Primary inference | The databases differed in overall publication volume but showed no statistically detectable difference in the shape of their annual publication trajectories. |

*Note. The primary model used k = 8. Alternative basis dimensions of k = 6 and k = 10 produced the same substantive conclusion. The nonsignificant difference smooth indicates no statistically detectable difference in trajectory shape; it does not establish database equivalence.*

# **Supplementary Figure S1. Diagnostic plots for the primary Poisson generalized additive model.**

# **Supplementary Figure S2. Residual autocorrelation functions for Web of Science and Scopus.**
